# The forces driving clonal expansion of the HIV-1 latent reservoir

**DOI:** 10.1186/s12985-019-1276-8

**Published:** 2020-01-07

**Authors:** Runxia Liu, Francesco R. Simonetti, Ya-Chi Ho

**Affiliations:** 1 Department of Microbial Pathogenesis, Yale University, New Haven, CT 06519 USA; 2 Department of Medicine, Johns Hopkins University, Baltimore, MD 21205 USA

**Keywords:** HIV-1 latent reservoir, Clonal expansion, Antigen-driven proliferation, Homeostatic proliferation, HIV-1 integration site, Aberrant proliferation, HIV-1 cure, HIV-1 proviral landscape, Defective HIV-1 proviruses, Chromatin accessibility

## Abstract

Despite antiretroviral therapy (ART) which halts HIV-1 replication and reduces plasma viral load to clinically undetectable levels, viral rebound inevitably occurs once ART is interrupted. HIV-1-infected cells can undergo clonal expansion, and these clonally expanded cells increase over time. Over 50% of latent reservoirs are maintained through clonal expansion. The clonally expanding HIV-1-infected cells, both in the blood and in the lymphoid tissues, contribute to viral rebound. The major drivers of clonal expansion of HIV-1-infected cells include antigen-driven proliferation, homeostatic proliferation and HIV-1 integration site-dependent proliferation. Here, we reviewed how viral, immunologic and genomic factors contribute to clonal expansion of HIV-1-infected cells, and how clonal expansion shapes the HIV-1 latent reservoir. Antigen-specific CD4^+^ T cells specific for different pathogens have different clonal expansion dynamics, depending on antigen exposure, cytokine profiles and exhaustion phenotypes. Homeostatic proliferation replenishes the HIV-1 latent reservoir without inducing viral expression and immune clearance. Integration site-dependent proliferation, a mechanism also deployed by other retroviruses, leads to slow but steady increase of HIV-1-infected cells harboring HIV-1 proviruses integrated in the same orientation at specific sites of certain cancer-related genes. Targeting clonally expanding HIV-1 latent reservoir without disrupting CD4^+^ T cell function is a top priority for HIV-1 eradication.

## Background

HIV-1 persists in the latent reservoir as a major barrier to cure [1–3]. CD4^+^ T cells harboring latent and transcriptionally inactive HIV-1 proviruses do not express viral antigens and do not die of viral cytopathic effects or immune clearance. While ART targets viral enzyme function or viral entry, ART does not affect HIV-1 transcription nor kills infected cells. Because of the extremely long half-life (~ 43–44 months) [4, 5] of the latent reservoir, it takes > 73 years for the latent reservoir to decay to zero [4]. Therefore, all HIV-1-infected individuals need to take life-long ART. There are 37 million people living with HIV-1 and only 62% of them requiring HIV-1 treatment have access to ART [6]. Given the adverse effects, economic burden and social stigma of life-long ART for the HIV-1-infected individuals, therapeutic strategies targeting the HIV-1 latent reservoir is required to end the HIV-1 endemic.

## Main text

### The HIV-1 latent reservoir undergoes clonal expansion

**Fig. 1:**
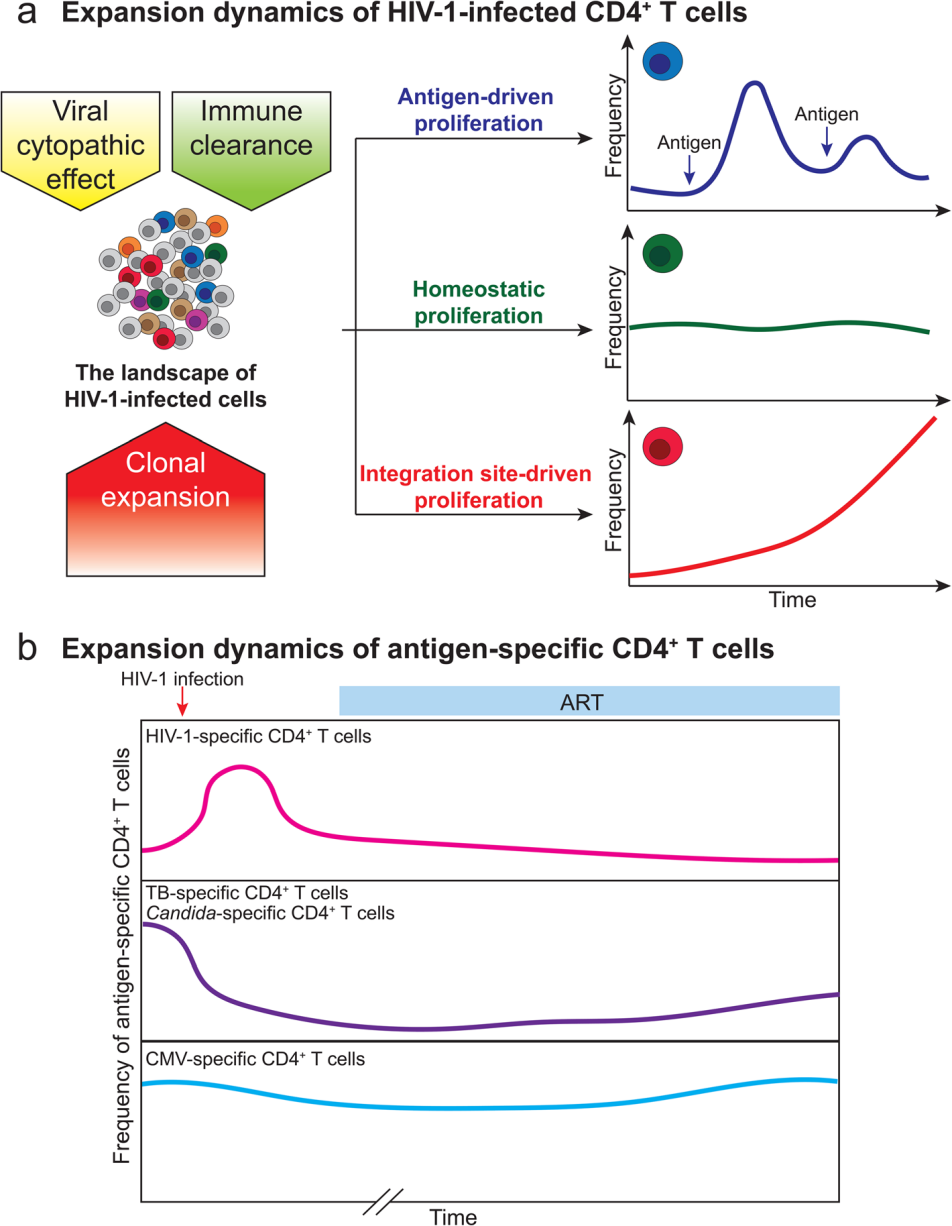
Expansion dynamics of HIV-1-infected CD4^+^ T cells during HIV-1 infection. **a** The landscape of HIV-1-infected cells is shaped by viral cytopathic effect, immune clearance and clonal expansion of the HIV-1-infected cells. The major drives of clonal expansion of HIV-1-infected cells include antigen-driven proliferation, homeostatic proliferation, and integration site-driven proliferation. HIV-1-infected antigen-specific cells surge as antigen stimulation peaks and wane as the antigen-specific response subsides. Homeostatic proliferation driven by cytokines such as IL-7 and IL-15 does not induce viral antigen expression and evades immune clearance. These two mechanisms are controlled by physiologic immune responses. In contrast, HIV-1 integration may drive aberrant cellular proliferation, which is not affected by host immune feedback controls. Thus, HIV-1 integration site-driven clonal expansion leads to a slow but steady increase of HIV-1-infected cells. Y axis, frequency of HIV-1-infected cells. **b** The clonal expansion dynamics of antigen-specific CD4^+^ T cells depends on antigen exposure, cytokine profiles and exhaustion phenotypes. HIV-1-specific CD4^+^ T cells increase during acute HIV-1 infection and decline after ART initiation as the majority of HIV-1 antigen is eliminated. Despite chronic antigen exposure, these HIV-1-specific CD4^+^ T cells are few, dysfunctional and impaired in proliferation capacity. On the other hand, TB-specific and *Candida*-specific CD4^+^ T cells are preferentially infected and depleted during HIV-1-infection, which can be partially restored upon ART. In contrast, CMV-specific CD4^+^ T cells are relatively protected from HIV-1 infection and remain relatively abundant and functional during HIV-1 infection

The landscape of the HIV-1-infected cells is shaped by viral cytopathic effects, immune clearance and clonal expansion of the infected cells (Fig. 1a). The size of the latent reservoir correlates with the area-under-the-curve of the product of viral load and CD4 count during acute infection, suggesting that reservoir seeding happens during peak viremia [7]. Indeed, early HIV-1-infection (within 4 weeks of expansion) can persist as clonally expanded HIV-1-infected cells [8]. However, it is the HIV-1-infected cells which are archived immediately before ART (which are likely survivors of ongoing immune selection pressure), as opposed to the initial peak viremia clones, which persist and undergo clonal expansion after years of ART [9, 10]. The persistence of HIV-1-infected cells does not mean that the same HIV-1-infected cells remain unchanged over the course of ART. HIV-1-infected cells undergo clonal expansion and the proportion of clonally expanded HIV-1-infected cells increase over time [11–13]. As > 90% of HIV-1 proviruses are defective [14–16], it was thought that these clonally expanded cells mainly harbor defective HIV-1 proviruses. However, three independent studies demonstrated that ~ 56% of cells harboring replication-competent HIV-1 proviruses undergo clonal expansion [17–19]. Similarly, HIV-1-infected cells in the lymphoid tissue undergo clonal expansion with no new rounds of ongoing replication under suppressive ART, as evidenced by the lack of phylogenetic evolution [10, 20, 21]. Considering that these observations are likely affected by under sampling (many clones are not large enough to be detected as expanded), these studies suggest that the majority of the latent reservoir are likely maintained by clonal expansion [17–19, 22]. Therefore, targeting the clonally expanding latently infected cells is a high-priority goal for HIV-1 eradication.

The major discrepancy in understanding HIV-1 clonal expansion dynamics is that the size of the HIV-1 latent reservoir does not change over time [4] but the cells that maintain this reservoir expand over time [17–19]. This indicates a major gap in understanding of clonal expansion dynamics during HIV-1-infection. We propose that 1) HIV-infected clones wax and wane in response to antigen stimulation, as part of the physiological immune responses of the host; 2) homeostatic proliferation induces expansion of HIV-1-infected cells without causing immune recognition and thus replenishes the latent reservoir; 3) HIV-1 integration site-dependent proliferation drives slow but steady increase of the infected cells (Fig. 1a).

### Clonally expanded HIV-1-infected CD4^+^ T cells in the peripheral blood and the lymphoid tissue contribute to viral rebound

There is considerable debate about which cellular subsets and anatomical compartments are the actual HIV-1 latent reservoir, and which of the reservoirs causes viral rebound during treatment interruption. To examine the sources of rebound viremia in vivo, analytical treatment interruption (ATI) were used in ART-suppressed, HIV-1-infected individuals [23]. By analyzing HIV-1 RNA sequences from limiting dilution viral outgrowth cultures and rebound plasma viruses after ATI, one study failed to find the identical matching HIV-1 sequences from the two sampling time points [24] while another study does [25]. Although the above study estimated the low contribution of HIV-1-infected cells in the peripheral blood as the major reservoir [26], multiple studies have shown that HIV-1-infected peripheral CD4^+^ T cells contribute to viral rebound [27–29]. First, activated HIV-1 proviruses by latency reversing agents from CD4^+^ T cells share identical sequence with the plasma viremia during ATI, indicating HIV-1-infected CD4^+^ T cells contribute to viral rebound [27]. Second, identical HIV-1 proviruses and cell-associated RNA sequences from clonally expended HIV-1-infected cells in the peripheral blood and in the lymphoid tissue on ART match the plasma RNA after ATI, suggesting in vivo clonally expanded CD4^+^ T cells in the peripheral blood and the lymphoid tissue are likely responsible for the viral rebound [28]. Third, a more comprehensive study showed various cell subsets and anatomical compartments including peripheral blood contribute to rebound viremia [29]. In individuals with larger clonally expanded HIV-1-infected cells in peripheral blood and lymphoid tissues, more identical sequences were found to match rebound plasma viruses, indicating the importance of clonal expansion in HIV-1 persistence and rebound dynamics [29].

### Expansion dynamics differ in HIV-1-infected CD4^+^ T cells harboring different subsets of proviruses

Despite ART, chronic immune activation persists in HIV-1-infected individuals [30, 31]. While ART blocks new rounds of infection to the neighboring cells, ART does not inhibit HIV-1 expression in the existing infected cells. Therefore, even under suppressive ART, the HIV-1 LTR promoter remains active, driving cell-associated HIV-1 RNA expression [32], production of viral particles and consequent T cell activation [33]. As both intact and defective HIV-1 proviruses may have intact HIV-1 promoter function [14], both intact and defective HIV-1 proviruses have the potential to express viral antigens upon stochastic reactivation [14, 34]. Further, as the frequency of defective proviruses (100–1000 per million CD4^+^ T cells) outnumbers the frequency of intact HIV-1 proviruses (1–100 per million CD4^+^ T cells) [14–16, 35], defective proviruses that can produce viral antigens will be an important source for chronic immune activation. The majority (> 90%) of HIV-1-infected proviruses are defective due to packaging signal deletions, large internal deletions, APOBEC3G-induced hypermutations and point mutations [14, 16, 34]. Using limiting dilution cell-associated RNA sequencing, it was shown that defective proviruses, such as those containing APOBEC3G-mediated hypermutations, are readily producing HIV-1 RNA without ex vivo stimulation [32]. In vitro analysis revealed that HIV-1 proviruses having packaging signal deletions can produce readily detectable levels of HIV-1 p24 antigen [14, 34]. Functional analysis revealed that these HIV-1 proviruses, despite having packaging signal deletions or inactivating APOBEC3G-mediated G-to-A hypermutations, can induce CD8^+^ T cell recognition [34]. Of note, large internal deletions seem to have dominant negative effect on viral protein production – that in proviruses with both hypermutations and large internal deletions, the HIV-1 proviruses will not be able to produce viral proteins and will not induce CD8^+^ T cell recognition of the infected cells [34]. While some proviruses with large internal deletions can activate alternative splice sites to produce spliced RNA products and potentially aberrant viral proteins [34, 36], the large internal deletions frequently encompass splice sites and splice elements and disables viral protein production [34, 37]. Therefore, CD4^+^ T cells harboring proviruses with large internal deletions are released from negative selective forces, and maybe preferentially expanded over time [16, 34]. These lines of evidence suggest that despite effective ART, HIV-1-infected cells, including those containing intact and defective proviruses, can continue to cause immune activation.

### Antigen stimulation drives dynamic expansion and contraction of HIV-1-infected cells

Clonal expansion of HIV-1-infected cells is driven by antigen-driven proliferation [38, 39], homeostatic proliferation [40, 41] and integration site-driven proliferation [11–13] (Fig. 1a). As HIV-1 proviruses reside in memory CD4^+^ T cells, it has been thought that the expansion dynamics of HIV-1-infected cells follows the physiologic expansion of memory CD4^+^ T cells by antigen-driven stimulation or cytokine-driven homeostatic proliferation (through interleukin (IL)-7 and IL-15). Indeed, in an HIV-1-infected individual who had uncontrolled metastatic squamous cell carcinoma, an HIV-1-infected CD4^+^ T cell clone expanded as the tumor progressed and contracted when cancer treatment was initiated [38]. Despite adherence to ART and the absence of drug-resistant viruses, plasma viral load surged as the tumor relapsed, suggesting that the expansion of the HIV-1-infected clone and HIV-1 expression were induced by a tumor-specific immune response. Elegant examination of this example of antigen-driven proliferation of HIV-1-infected cells provides insights into some previously unexplained clinical scenarios, such as the presence of viral blips and predominant plasma clones despite ART. First, in HIV-1-infected individuals adherent to ART, clinically detectable levels of plasma viremia can still be occasionally captured. Such intermittent low-level viremia (plasma viral load < 200 copies/ml), termed viral blips, is devoid of drug resistance mutations, does not benefit from treatment intensification, and does not require changes in antiretroviral regimens [42]. Phylogenetic analysis during episodes of low-level viremia revealed genetically identical viruses termed the predominant plasma clones [43–45]. Based on the antigen-driven HIV-1-infected T cell clonal expansion dynamics, it is likely that antigen stimulation activates HIV-1-infected, antigen-specific CD4^+^ T cells and drives HIV-1 expression and clonal expansion. Thus, the predominant plasma clones which wax (during antigen stimulation) and wanes (when antigen stimulation resolves) over time [46]. While concurrent ART remains effective in preventing ongoing HIV-1 replication, ART does not inhibit HIV-1 LTR promoter function, viral RNA expression or clonal expansion of the HIV-1-infected cells. Such antigen-driven proliferation of HIV-1-infected cells is likely not integration site dependent – that HIV-1 integration sites in these proliferated cells, likely driven by antigen stimulation, are typically not in specific cancer-related genes (see below) [38, 47]. These HIV-1-infected, antigen-specific CD4^+^ T cells undergo HIV-1 expression and clonal expansion, leading to transient residual viremia and viral blips [47]. Thus, antigen stimulation-induced viral blips are typically transient, which surges as antigen stimulation peaks and wanes as the antigen-specific response subsides. However, in depth characterization of nine individuals with residual viremia caused by expanded clones carrying replication-competent proviruses, showed long periods of stable or intermittent viral production (median 3.2 years) [47], suggesting that in some cases the response to certain antigenic stimulations may persist over time.

### Expansion dynamics differ in HIV-1-infected CD4^+^ T cells specific for different pathogens

The expansion dynamics of HIV-1-infected cells differ between CD4^+^ T cells specific for different antigens (Fig. 1b). HIV-1-specific CD4^+^ T cells are required for HIV-1 control [48]. Presumably both HIV-1-infected CD4^+^ T cells and professional antigen presenting cells can provide constant immune activation to HIV-1-specific CD4^+^ T cells and induce HIV-1-specific CD4^+^ T cell proliferation. The HIV-1-infected cells are enriched in memory cells polarized in Th1 [49] or expressing effector memory phenotypes [50]. While HIV-1-specific CD4^+^ T cells are readily detected in treated and untreated HIV-1-infected individuals [51], these HIV-1-specific T cells are few, dysfunctional and impaired in proliferation capacity [52, 53], due to T cell activation [54], chronic immune activation [55], upregulation of inhibitory molecules [56–58], and the loss of lymphoid structure supporting CD4 homeostasis [59–61] (Fig. 1a). While HIV-1 preferentially infects HIV-1-specific cells in the context of acute and recrudescent HIV-1 infection [39], cytopathic effects [62] may lead to clonal depletion of HIV-1-infected cells. Early ART, which halts ongoing immune activation and new rounds of viral infection, restores the frequency and proliferative responses of HIV-1-specific CD4^+^ T cells compared to untreated individuals [63]. Therefore, due to the complexity of ongoing antigen stimulation (which drives proliferation) and immune exhaustion (which reduces proliferation capacity), it remains to be determined how HIV-1-specific CD4^+^ T cells, and the HIV-1 proviruses which reside in them, expand or contract over the course of HIV-1 infection, before and after ART introduction.

The difference in susceptibility of clonal depletion is potentially due to the cytokine profiles of the pathogen specific CD4^+^ T cells (Fig. 1b). Similar to HIV-1-specific CD4^+^ T cells, *Mycobacterium tuberculosis* (TB)-specific CD4^+^ T cells are preferentially depleted early during HIV-1 infection due to viral cytopathic effect and the loss of proliferation capacity due to chronic immune activation [64]. TB-specific CD4^+^ T cells have increased expression of CCR5 and produce IL-2 and IL-2 receptor CD25 [64, 65]. Binding of IL-2 to CD25 promote cellular proliferation and HIV-1 replication. Thus, TB-specific CD4^+^ T cells are preferentially infected and depleted by HIV-1 infection. After ART, TB-specific CD4^+^ T cells can be restored [66]. Similarly, *Candida albicans*-specific CD4^+^ T cells are also preferentially infected by HIV-1 and depleted during progressive HIV-1 infection [67]. *Candida* specific-CD4^+^ T cells express more IL-2, IL-17 and CD25 and are highly susceptible to HIV-1 infection. Candida specific-CD4^+^ T cells are preferentially lost at early HIV-1 infection with ongoing CD4 depletion [67]. In contrast, cytomegalovirus (CMV) specific CD4^+^ T cells are preserved in function, quantity and proliferation capacity during HIV-1 infection [68–70]. CMV-specific CD4^+^ T cells express lower level of PD-1 than HIV-1-specific CD4^+^ T cells [57, 71]. The cytokine profile of CMV-specific CD4^+^ T cells provide survival benefit during HIV-1-infection. For example, CMV-specific CD4^+^ T cells express high levels of MIP-1β while TB-specific CD4^+^ T cells do not [65]. MIP-1β binds to and downregulates its ligand CCR5, preventing HIV-1 infection [72]. Further, CMV-specific CD4^+^ T cells produce CD57, a marker for limiting proliferation, which restricts HIV-1 replication [73, 74]. Thus, CMV-specific CD4^+^ T cells are less susceptible to HIV-1 infection and are preserved. During latent CMV infection, consistent low level of antigen stimulation maintains memory inflation of short-lived, functional CMV-specific T cells [75]. Thus, CMV-specific CD4^+^ T cells remains relatively functional during HIV-1 infection. CMV-specific CD4+ T cells, if infected with HIV-1 (although less susceptible), may proliferate at a higher rate due to intermittent CMV antigen stimulation and the retained proliferation capacity.

### HIV-1-infected cells evade immune clearance through IL-7-driven homeostatic proliferation

Homeostatic proliferation maintains the repertoire of memory CD4^+^ T cells [76–78]. During chronic HIV-1-infection, the proliferation capacity of CD4^+^ T cells is significantly impaired because of decreased IL-7 receptor expression [79], chronic immune activation [80], immune exhaustion [58, 81, 82], and the destruction of lymphoid tissue [83]. IL-7 expression level is upregulated in response to CD4^+^ T cell depletion during HIV-1-infection [84], promoting proliferation of HIV-1-infected CD4^+^ T cells. Interestingly, IL-7 induces proliferation of HIV-1-infected cells without reactivating latent HIV-1 [85, 86], suggesting that HIV-1-infected CD4^+^ T cells may undergo homeostatic proliferation without being recognized by immune surveillance.

### Retroviral integration into cancer-related genes promotes clonal expansion

While HIV-1 does not cause cancer in the infected cell, many retroviruses induce insertional oncogenesis and uncontrolled clonal expansion of the infected cell. For example, the discovery of oncogene originates from research on retroviral pathogenesis. Rous sarcoma virus is the first retrovirus that was discovered and known to cause cancer in its avian host, leading to the discovery of oncogenes [87]. Lessons about retroviral-induced insertional oncogenesis in humans were learned from therapeutic retroviral vectors and human T lymphotropic virus (HTLV) infections.

Retroviral vectors have been used as a gene therapy tool to correct genetic diseases. For example, individuals with X-linked severe combined immunodeficiency (SCID-X1) were treated by gene therapy to restore interleukin receptor γ gene in bone marrow CD34^+^ precursor cells using gammaretroviral vectors [88]. However, four out of the nine patients who received gene therapy developed T cell leukemia, due to the gammaretroviral vectors insertion-mediated activation of proto-oncogenes, such as *BMI1* and *CCND2* or disruption of tumor suppressor genes such as *CDKN2A*, resulting into uncontrolled T cells growth [89]. Such Moloney murine leukemia virus (MLV)-based gene therapy induces leukemia in treated patients, likely due to MLV preferentially integrating into the transcription start sites [90].

Understanding retroviral insertional oncogenesis led to the use of safer, non-oncogenic retroviral vectors such as lentiviruses. In an example of lentiviral vector mediated gene therapy for β-thalassemia, the lentiviral vector encoding β-globin integrated in the same orientation of the transcription regulator *HMGA2* gene, disrupted HMGA2-mediated transcriptional regulation, and caused clonal expansion of this T cell clone [91]. In another example, lentiviral vectors carrying the chimeric antigen receptor (CAR) cassette in the treatment of chronic lymphocytic leukemia integrated into the intron of the tumor suppressor gene *TET2*, disrupted TET2 regulatory region and led to a dominant clone (94% at the peak of response) in vivo [92]. This suggests that non-oncogenic lentiviruses can induce clonal expansion of the transduced primary T cells in vivo.

HTLV, the first reported human oncogenic retrovirus causes adult T cell lymphoma-leukemia (ATL) [93, 94]. While HTLV causes cancer through several mechanisms regardless of the integration site, such as viral *HBZ* mRNA transcription and protein Tax, HTLV interaction with the host chromatin at the integration site is a major mechanism for oncogenesis (reviewed in [95]). Unlike HIV-1, HTLV has CTCF binding sites within the proviral genome, which allows distant host gene interactions through CTCF-mediated chromatin looping [96]. While initial integration does not favor specific chromosomes, HTLV integration into acrocentric chromosomes provides a higher survival benefit [97]. Similar to HIV-1, HTLV integration preferentially occurs at actively transcribed genes [98]. The host genomic environment at HTLV integration site determines HTLV clonal expansion in vivo and favors insertions with the same orientation as the nearest host gene [98]. Thus, over the scale of 50–60 years, a dominant clone grows out of host control and leads to ATL. Given the similarity between HIV-1 and HTLV induced clonal expansion in the infected lymphocytes, further examination of mechanisms of HIV-1-induced clonal expansion may provide therapeutic targets to disrupt HIV-1-driven clonal expansion without damaging the uninfected cells.

### Integration site-dependent proliferation drives the proliferation of HIV-1-infected cells

HIV-1 preferentially integrates into introns of actively transcribed genes, both in vitro and in vivo [14, 99–101]. In these studies, HIV-1 integration sites were identified but at a small scale [100]. Using modified deep sequencing approaches to examine and HTLV integration sites developed by the Bangham group [98], thousands of HIV-1 integration sites in HIV-1-infected individuals were identified for a more comprehensive examination of the HIV-1 integration landscape [11]. Despite that HIV-1integration into T cell genomes is biased by multiple viral and host factors (CPSF6 [102] and LEDGF/p75 [103]), cells harboring HIV-1 proviruses which are integrated into the exact same nucleotide is unlikely to come from two distinct integration events. Rather, it is more likely the result of one infection event followed by proliferation of the infected cells. Therefore, HIV-1 proviruses having the exact same integration site indicates clonal expansion of the infected cells. Using sonication-based random DNA shearing, the same HIV-1 integration site with different DNA shearing breakpoints indicates the number of cells that belong to the same clone. This method, called sonic abundance [104], identifies both the integration site and the number of clonally expanded HIV-1-infected cells. These integration site analyses revealed dramatic difference of HIV-1-integration landscape in vitro versus in vivo. First, the frequency of HIV-1 integration into cancer-related genes (12.5%) in HIV-1-infected individuals is significantly higher than the frequency of cancer-related genes in the human genome (5.19%) [12]. Second, the integration patterns in vivo and in vitro are strikingly different. During in vitro infection, HIV-1-integration sites are relatively random throughout the introns of genes, both in the same and opposite orientation in respect to the host transcription unit [11, 12]. However, during in vivo infection in CD4^+^ T cells from virally suppressed HIV-1-infected individuals, HIV-1 integration sites are enriched in a small region in certain cancer-related genes, such as in the introns immediately upstream the translation start site of cancer-related genes *BACH2*, *MKL*2 and *STAT5B* [11, 12]. In addition, HIV-1 proviruses are integrated exclusively in the same orientation with the host transcription unit at these sites, which is the opposite of what happens in vitro (that HIV-1 integration into the same and opposite orientation is roughly equal [101]). HIV-1 integration into specific sites associated with clonal expansion in vivo, such as *BACH2*, *MKL2*, *NFATC3* and *STAT5B*, have been captured in multiple studies, using different methods in different HIV-1-infected individuals [11, 12, 105, 106]. These specific sites recur across individuals not because of preferential integration, as HIV-1 integration into these sites are not enriched during in vitro infections [11]. Similar to HIV-1 integration sites, simian immunodeficiency virus (SIV) with integration into *BACH2*, *MKL2* and *STAT5B* are found in SIV-infected macaques before ART [107]. Despite that the genome-wide distribution of HIV-1 and SIV integration showed a high degree of overlap in vitro, it seems that more integrants are oriented in the convergent orientation of these genes in SIV-infected macaques under suppression, which is opposite from what observed from ART treated HIV-1-infected individuals in vivo [11, 12, 107]. However, more SIV integration site data from long-term treated macaques are needed to determine whether there is positive selection of SIV proviruses integrated in genes associated with clonal expansion in individuals on ART. Nevertheless, the specific mechanisms driving HIV-1 integration site-dependent proliferation, which happens in vivo but not in vitro, remain unclear.

In some instances, these drives (antigen-driven proliferation, homeostasis-driven proliferation and integration site-driven proliferation) of clonal expansion may act together. HIV-1-infected CMV-specific CD4^+^ T cells may inflate due to consistent CMV antigen stimulation at late stage of CMV infection [75]. CD127 (IL-7 receptor) are highly expressed on inflationary CMV-specific CD8^+^ T cells [108] and may presumably be expressed on CMV-specific CD4^+^ T cells. If HIV-1 provirus happens to integrate into cancer-related genes, such as *BACH2* and *MKL2*, the infected cells may undergo aberrant proliferation [11, 12]. All these factors could promote the proliferation of HIV-1-infected cells.

**Fig. 2:**
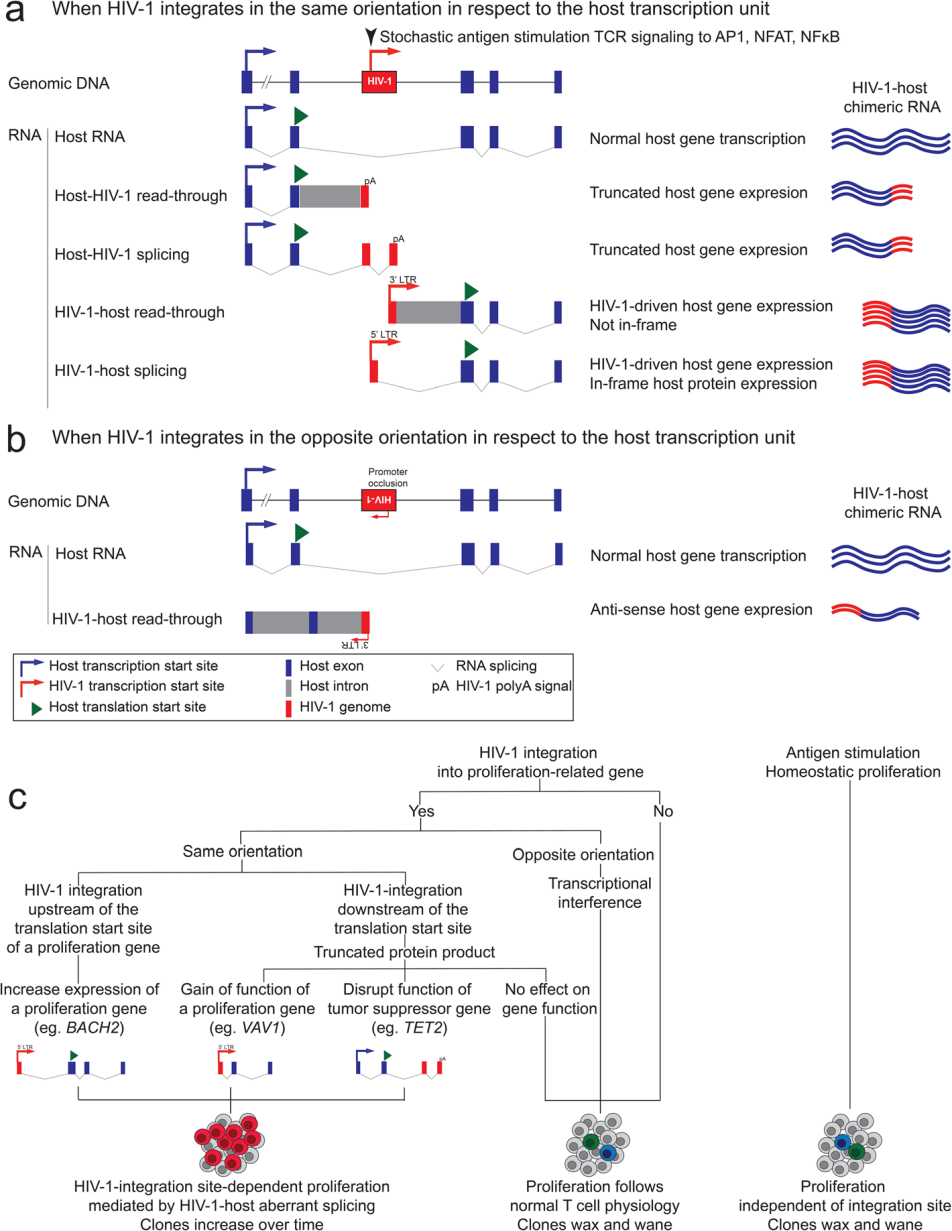
Mechanisms of integration site-dependent clonal expansion of HIV-1-infected cells. HIV-1-host interactions at the integration site when HIV-1 is integrated in the same (**a**) or opposite (**b**) orientation in respect to the transcription unit. **c** HIV-1-driven integration site-dependent proliferation depends on the orientation, orientation and the functional consequences of the host gene in which HIV-1 is integrated in

### HIV-1 proviruses which are integrated into specific cancer-related genes can be intact

Whether clonally expanded HIV-1 proviruses in these specific sites of cancer-related genes are intact or defective was unknown. Since over 90% of HIV-1 are defective [14–16], based on the possibility, the majority of clonally expanded cells should harbor defective HIV-1 proviruses [13]. However, it remains technically challenging to examine HIV-1 integration site and HIV-1 genome integrity at the same time in a high throughput way to examine the integration site landscape of replication competent HIV-1. First, when using random shearing for HIV-1 integration site analysis, the HIV-1 genome is disrupted, preventing simultaneous examination of HIV-1 integration site and HIV-1 genome integrity at the same time [11–13]. Second, in viral outgrowth experiments trying to capture the clonality of replication competent HIV-1, cells in the viral outgrowth cultures underwent multiple rounds of in vitro infection, and HIV-1 integration sites captured in the culture wells cannot reflect HIV-1 integration sites in vivo [17–19]. Third, full-length HIV-1 proviral sequencing methods, which can capture clonally expanded HIV-1, amplifies regions spanning HIV-1 genome and excludes integration site information [14, 16].

In response to this challenge, several methods were developed to examine HIV-1 integration site and HIV-1 genome integrity at the same time. First, using whole genome amplification by phi29 polymerase, the Lichterfeld group [109] and the Kearney group [110] developed matched integration site and proviral sequencing to examine the integration site and HIV-1 near-full length genome sequencing at the same time. Second, using limiting dilution culture and CD3/CD28-mediated proliferation, the Siliciano group sequenced the HIV-1 integration site and HIV-1 near full-length genome from CD4^+^ T cells undergoing ex vivo proliferation. Of note, cells harboring replication competent HIV-1 died of viral cytopathic effects in this study after 3 weeks of maximum T cell activation, and only defective proviral clones were identified. The HIV-1 proviruses integrated into the cancer-related gene *BACH2* (2 clones total) from these two methods are defective. However, since both methods attempts to examine all HIV-1 proviruses, the majority of the integration sites captured are from defective proviruses, and the number of integration sites of intact HIV-1 remain limited to draw conclusions. The fact that over 50% cells harboring infectious HIV-1 proviruses are from clonal expansion [17–19] suggests that other methods which can preferentially enrich for intact HIV-1 are needed to examine the HIV-1 integration site landscape of replication competent proviruses. Our group developed HIV-1 Sortseq which identifies HIV-1-infected cells expressing readily detectable levels of HIV-1 RNA [111]. Using HIV-1-chimeric RNA junction analysis, we identified cells which harbor inducible HIV-1 integrated into cancer-related genes found in clonally expanded cells in vivo, such as *BACH2* and *NFATC3.* Thus, both intact and defective HIV-1can be integrated into cancer-related genes, and both intact and defective HIV-1 proviruses can undergo clonal expansion. As the landscape of HIV-1 integration is heterogeneous, thus it is difficult to draw conclusions. Finding defective proviruses integrated into recurrent integration genes such as *BACH2* does not indicate that all HIV-1 integrated into *BACH2* are defective. Similarly, finding clonally expanded cells integrated into non-cancer related genes does not indicate that HIV-1 integration into cancer-related genes does not cause clonal expansion. A more high-throughput method which can break the technical barrier (that 90% of the sequences or proviruses isolated are defective) and detect HIV-1 integration sites of intact HIV-1 proviruses is necessary to understand HIV-1 integration site-dependent clonal expansion mechanisms.

### Mechanisms of integration site-dependent proliferation

The majority of HIV-1 proviruses are integrated into the introns of actively transcribed genes [100]. HIV-1 can be integrated into the host transcription unit in the same (Fig. 2a) or opposite (Fig. 2b) orientation. When HIV-1 is integrated in the same orientation, the host and the HIV-1 promoter compete for the RNA polymerase and the transcription machinery, creating transcriptional interference. Transcriptional interference is typically thought as a mechanism that the host gene expression suppresses HIV-1 gene expression through viral promoter occlusion [112, 113] (Fig. 2a and b). For HIV-1 proviruses integrated in the same orientation as the host transcription unit (Fig. 2a), transcription from host gene leads to readthrough transcription into HIV-1 provirus or transcriptional termination at the HIV-1 polyA signal [113]. For HIV-1 proviruses integrated in the opposite orientation as the host transcription unit (Fig. 2b), viral promoter occlusion reduces the level of HIV-1 transcription [112].

Upon T cell activation, such as antigen stimulation which signals through T cell receptor pathways, transcription factors AP1, NFAT and NFκB translocate into the nucleus, bind to the respective binding sites on HIV-1 promoter and lead to stochastic HIV-1 activation. Such T cell activation relieves the aforementioned host-mediated transcriptional interference and allows HIV-1-driven transcription [113]. Therefore, upon stimulation, for HIV-1 proviruses integrated in the same orientation as the host transcription unit, HIV-1 promoter drives HIV-1 transcription and host gene expression through HIV-1-to-host RNA splicing (Fig. 2a, see below) [106, 111]. For HIV-1 proviruses integrated in the opposite orientation as the host transcription unit, HIV-1 3′ LTR can drive anti-sense host RNA transcription and can potentially interfere with normal host gene transcription [111] (Fig. 2b).

When HIV-1 dominates over the host promoter upon stochastic activation, HIV-1 promoter drives aberrant host gene transcription. This means that the host gene expression is controlled by HIV-1 promoter activity not under cellular regulation. Detailed analysis on HIV-1-host RNA splicing revealed the importance of HIV-1-driven aberrant host gene expression at the integration site as a mechanism for integration site-dependent proliferation. Upon stochastic activation, HIV-1 promoter drives HIV-1 transcription and viral RNA production. Typically, HIV-1 RNA splices from HIV-1 splice donors (such as the major splice donor) to HIV-1 splice acceptors and produces spliced HIV-1 RNA. However, HIV-1 RNA can also splice from a HIV-1 splice donor into a host splice acceptor [106, 111, 113, 114] (Fig. 2a). Therefore, when HIV-1 is integrated upstream of the host gene translation start site, such as *BACH2*, *MKL2* and *STAT5B* [11, 12, 106], HIV-1 promoter drives HIV-1 transcription and induces RNA splicing from HIV-1 major splice donor into the host gene splice acceptor, and leads to transcription of the full coding sequence of the host gene, such as in the proliferation-related gene *BACH2* [106, 111]. When HIV-1 is integrated into a proliferation-related gene downstream of the translation start site, such as the proto-oncogene *VAV1*, HIV-1 interrupts into the middle of the VAV1 coding sequencing, leading to N-terminal truncated VAV1 protein expression. As N-terminal VAV1 truncation removes the regulatory region of VAV1, this HIV-1-driven truncated VAV1 expression leads to increased cellular proliferation [111] (Fig. 2c). A similar example in lenviral transduction for chimeric antigen receptor (CAR)-T cell editing, a lentiviral insertion into a tumor suppressor gene *TET2* downstream of the host gene translation start site leads to host-to-lentiviral splicing into the lentiviral genome and transcriptional termination, leading to C-terminal truncation of the tumor suppressor gene *TET2* expression and increased proliferation of the T cell clone [92] (Fig. 2c).

HIV-1 integration into cancer-related gene alone does not determine integration site-dependent proliferation (Fig. 2c). First, it depends on the location and direction of the integration event [11, 12]. Second, it depends on whether the resulting HIV-1-induced aberrant host gene transcription induces a significant change in the gene expression and function, such as increased proliferation-related gene expression (such as *BACH2*), gain-of-function truncation in a proliferation-related gene (such as *VAV1*), or loss-of-function truncation in a tumor suppressor gene (*TET2)*. Of note, in overt T cell activation, such as antigen-driven proliferation and homeostatic proliferation, the proliferation of the infected cell does not depend on the HIV-1 integration site. Clonally expanded cells can still be captured in antigen-induced proliferation harboring HIV-1 integrated into sites irrelevant to proliferation [38]. The difference is that while antigen stimulation follows host immune homeostasis control and the HIV-1-infected clones may wane upon antigen removal, HIV-1-driven integration site-dependent proliferation will gradually increase over time (Fig. 2c), although such increase may take a scale of years of in vivo selection to be observed [11, 12].

### HIV-1 integration site-dependent clonal expansion – does the chromatin environment matter?

The integration sites that are found repeatedly, in vivo but not in vitro, are associated with integration site-driven proliferation [11, 12, 105]. These genes are termed “recurrent integration genes” [115]. While HTLV mediates chromatin looping through CTCF sites within the HTLV genome and changes the enhancer landscape, HIV-1 proviruses do not have CTCF sites to similarly alter chromatin structure [96]. Still, researchers hypothesize that local chromatin environment contributes to clonal expansion only when HIV-1 proviruses are integrated in these recurrent integration sites. For example, in an in vitro model, it was proposed that these recurrent integration genes are located near the nuclear pore where HIV-1 integration occurs [115, 116]. These recurrent integration genes are spatially clustered during T cell activation and proximal to super-enhancers [115]. By mapping the HIV-1 integration sites at the recurrent integration genes with a separate dataset of CD4^+^ T cell chromatin accessibility landscape using Assay for Transposase-Accessible Chromatin using sequencing (ATACseq), it seems like these recurrent integration genes have more accessible chromatin region near these HIV-1 integration sites, and therefore potentially contributes to clonal expansion. However, testing this hypothesis in CD4^+^ T cells from HIV-1-infected individuals remain challenging due to the rarity of HIV-1-infected cells and the lack of selection markers to identify these cells. In contrast, overlaying HIV-1 integration sites and ATACseq (from a separate aliquots of CD4^+^ T cells from the same individual) from three HIV-1-infected individuals suggests that HIV-1 proviruses may integrate into loci away from accessible regions [109]. Nevertheless, examination of chromatin accessibility at the HIV-1 integration site remains technically not possible, and whether the chromatin environment at the HIV-1 integration sites favors clonal expansion or prevents gene expression remains under debate.

## Conclusions

While antigen-driven proliferation and homeostatic proliferation are under host immune regulation, HIV-1 integration site-driven proliferation is not inhibited by host immune feedback controls. Therefore, clones driven to expand by the effect of HIV-1 integration may accumulate over time, similar to how HTLV causes leukemia. While it takes 50–60 years for HTLV to induce cancer transformation of the infected cell, HIV-1 does not eventually cause cancer in the infected cell. Still, proliferation of HIV-1-infected cells through HIV-1-driven proliferation is a major mechanism of HIV-1 persistence. Targeting the proliferating HIV-1-infected cells without disrupting normal CD4^+^ T cell function is a top priority to eliminate the clonally expanding HIV-1 reservoir. For example, ongoing clinical trials are investigating whether inhibition of T cell proliferation can accelerate the decay of the latent reservoir (NCT03262441) [117]. Since homeostatic proliferation does not induce HIV-1 antigen expression, immune therapies requiring HIV-1 protein expression, such as broadly neutralizing antibodies, may not affect this expanding reservoir unless combined with strong reversal of HIV-1latency. Strategies targeting proliferation of HIV-1-infected cells, but not uninfected cells, should be searched to eliminate the clonally expanding latent reservoir.

## Data Availability

Not applicable.

## Abbreviations

ART: Antiretroviral therapy
ATACseq: Assay for Transposase-Accessible Chromatin using sequencing
ATI: Analytical treatment interruption
ATL: Adult T cell lymphoma-leukemia
CAR: Chimeric antigen receptor
CMV: Cytomegalovirus
HIV-1: Human immunodeficiency virus type 1
HTLV: Human T lymphotropic virus
IL: Interleukin
MLV: Moloney murine leukemia virus
SCID-X1: X-linked severe combined immunodeficiency
SIV: Simian immunodeficiency virus
TB: *Mycobacterium tuberculosis*
